# Ventricular tachycardia ablation in late-onset phenotype Fabry disease: a case report

**DOI:** 10.1093/ehjcr/ytag746

**Published:** 2026-10-12

**Authors:** Khuong Dang Tran, Hendyono Lim, Phuoc Huu Nguyen, Chin-Yu Lin

**Affiliations:** Division of Cardiac Electrophysiology, Cardiovascular Center, Taipei Veterans General Hospital, No. 201, Sec. 2, Shipai Rd., Beitou District, Taipei 11217, Taiwan; Heart Rhythm Unit, Cardiology Department, University Medical Center Ho Chi Minh City, 215 Hong Bang Street, Cho Lon Ward, Ho Chi Minh City 72723, Vietnam; Division of Cardiac Electrophysiology, Cardiovascular Center, Taipei Veterans General Hospital, No. 201, Sec. 2, Shipai Rd., Beitou District, Taipei 11217, Taiwan; School of Medicine, College of Medicine, National Yang Ming Chiao Tung University, No. 155, Section 2, Linong Street, Beitou District, Taipei 112304, Taiwan; Diagnostic Imaging Department, University Medical Center HCMC, 215 Hong Bang St., Ho Chi Minh City 700000, Vietnam; Division of Cardiac Electrophysiology, Cardiovascular Center, Taipei Veterans General Hospital, No. 201, Sec. 2, Shipai Rd., Beitou District, Taipei 11217, Taiwan; School of Medicine, College of Medicine, National Yang Ming Chiao Tung University, No. 155, Section 2, Linong Street, Beitou District, Taipei 112304, Taiwan

**Keywords:** Fabry disease, Ventricular tachycardia, Catheter ablation, Case report

## Abstract

**Background:**

Fabry disease is an X-linked lysosomal storage disorder that can cause progressive cardiomyopathy, myocardial fibrosis, conduction disease, and malignant ventricular arrhythmias. Ventricular tachycardia (VT) ablation in advanced Fabry cardiomyopathy is challenging, particularly in patients with extensive scar and left ventricular assist device (LVAD) support.

**Case summary:**

A 71-year-old man with late-onset Fabry disease, confirmed by endomyocardial biopsy showing myelin body accumulation, reduced α-galactosidase A activity, and elevated plasma Gb3, developed severe left ventricular dysfunction, extensive late gadolinium enhancement, LVAD implantation, and complete atrioventricular block requiring dual-chamber pacemaker implantation. He presented with recurrent palpitations and near-syncope. Device interrogation revealed multiple VT episodes, including one episode degenerating into ventricular fibrillation, and surface electrocardiography documented wide-complex tachycardia with right bundle branch block morphology and an inferior axis. An electrophysiological study was performed using an antegrade transseptal left ventricular approach. Intracardiac echocardiography was used to delineate the LV cavity and LVAD position, facilitating safe catheter navigation and mapping while avoiding interference with the LVAD. Endocardial mapping demonstrated diffuse late potentials, low-voltage substrate, and deceleration zones involving the basal-to-mid-inferolateral left ventricle. Substrate modification targeting these abnormal electrograms eliminated inducible ventricular premature complexes. No recurrent VT episodes were detected on subsequent pacemaker interrogation.

**Discussion:**

This case highlights the complex arrhythmogenic substrate of late-stage Fabry cardiomyopathy and the utility of intracardiac echocardiography-guided mapping in patients with LVAD support. Catheter ablation may provide short-term arrhythmia control, but in advanced Fabry disease, its role remains largely palliative.

Learning points

- Ventricular tachycardia ablation may provide acute arrhythmia control in advanced Fabry disease, but remains largely palliative.
- In patients with left ventricular assist device support, intracardiac echocardiography may enable safer left ventricular mapping by delineating the inflow cannula.

## Introduction

Ventricular arrhythmia (VA) is a significant complication in patients with Fabry disease (FD), a rare X-linked lysosomal storage disorder caused by deficiency in the enzyme α-galactosidase A. This enzyme deficiency leads to the accumulation of glycosphingolipids, particularly in the heart, causing various arrhythmias, including VA, bradyarrhythmia, and atrial fibrillation.^1,2^ The pathophysiology of VA in FD involves several stages of cardiomyopathy, including glycosphingolipid accumulation, left ventricular hypertrophy, inflammation, and fibrosis. Implantable cardioverter-defibrillator (ICD) implantation is recommended for secondary prevention in patients with VA, resulting in aborted sudden cardiac death (SCD) or unexplained syncope, and may also be considered in selected high-risk patients with advanced left ventricular hypertrophy and extensive myocardial fibrosis.^3^ Although contemporary VA guidelines provide general recommendations for the management of sustained VA in structural heart disease, robust disease-specific guidance for catheter ablation in Fabry cardiomyopathy remains limited.^4^ Previous reports suggest that ventricular tachycardia (VT) in FD is often scar related and may be amenable to substrate-based catheter ablation; however, the procedure can be technically challenging because the arrhythmogenic substrate may be extensive, intramural, or epicardial.^5^ We present a patient with late-onset FD with recurrent VT.

## Summary figure

**Figure:**
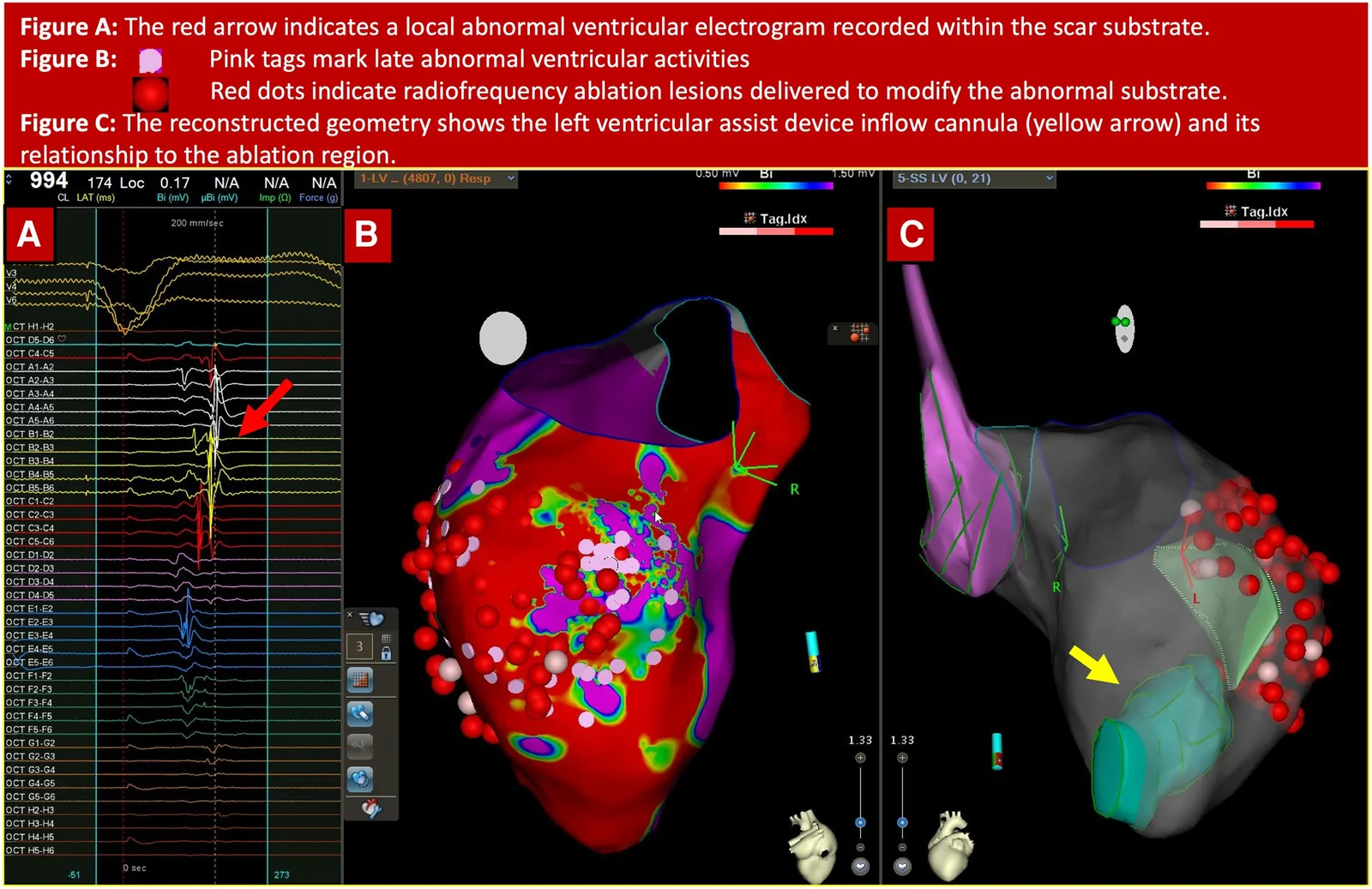


## Case presentation

A 71-year-old male presented with episodes of palpitations and near-syncope. He reported exertional dyspnoea corresponding to New York Heart Association functional Class II. He had been diagnosed with FD in 2017, which was confirmed by an endomyocardial biopsy showing cardiomyocytes with myelin body accumulation and mild interstitial fibrosis. Cardiac magnetic resonance (CMR) imaging showed left ventricular (LV) hypertrophy, LV enlargement, and global hypokinesia, with extensive late gadolinium enhancement (LGE) in a mixed distribution. Late gadolinium enhancement involved the basal-to-mid-septal segments and right ventricular (RV) insertion sites in a mid-wall pattern, the basal-to-mid-anterior/anterolateral LV in a limited subendocardial pattern, and the apical anterior, lateral, and inferior walls in a nearly transmural pattern (*Figure 1*). Together with markedly reduced α-galactosidase A activity (1.0% of normal), elevated plasma globotriaosylceramide (Gb3) (6.0 µg/mL; reference <4.2 µg/mL), elevated plasma lyso-Gb3 (6.0 nM; reference < 2.6 nM), and genetic testing showing a pathogenic GLA mutation, these findings confirmed the diagnosis of FD. Coronary angiography demonstrated no significant epicardial coronary artery stenosis. On initial CMR imaging in 2017, the left ventricular ejection fraction (LVEF) was 41%, and the left ventricular end-diastolic dimension was 75 mm; then, the LVEF subsequently declined to 27%. Because of progressive heart failure with reduced ejection fraction (HFrEF), the patient underwent implantation of a left ventricular assist device (LVAD; HeartMate III) in March 2019, after which the LVEF partially recovered to ∼36%. In 2022, he was diagnosed with complete atrioventricular (AV) block, necessitating dual-chamber pacemaker implantation. A dual-chamber pacemaker, rather than a cardiac resynchronization therapy-defibrillator (CRT-D), was implanted at that time because the patient had an active LVAD driveline infection; a smaller device was therefore chosen to minimize the risk of device-related infection with a CRT-D upgrade planned at a later stage. Heart transplantation had been considered; however, he was not a candidate because of advanced age. The patient was receiving amiodarone 200 mg daily for recent non-sustained VA, together with HFrEF therapy, including sacubitril/valsartan and dapagliflozin. A beta-blocker was not added because of severe chronic obstructive pulmonary disease requiring long-acting beta-agonist bronchodilator therapy. He received enzyme replacement therapy (ERT) four times between December 2018 and March 2019.

**Figure 1:**
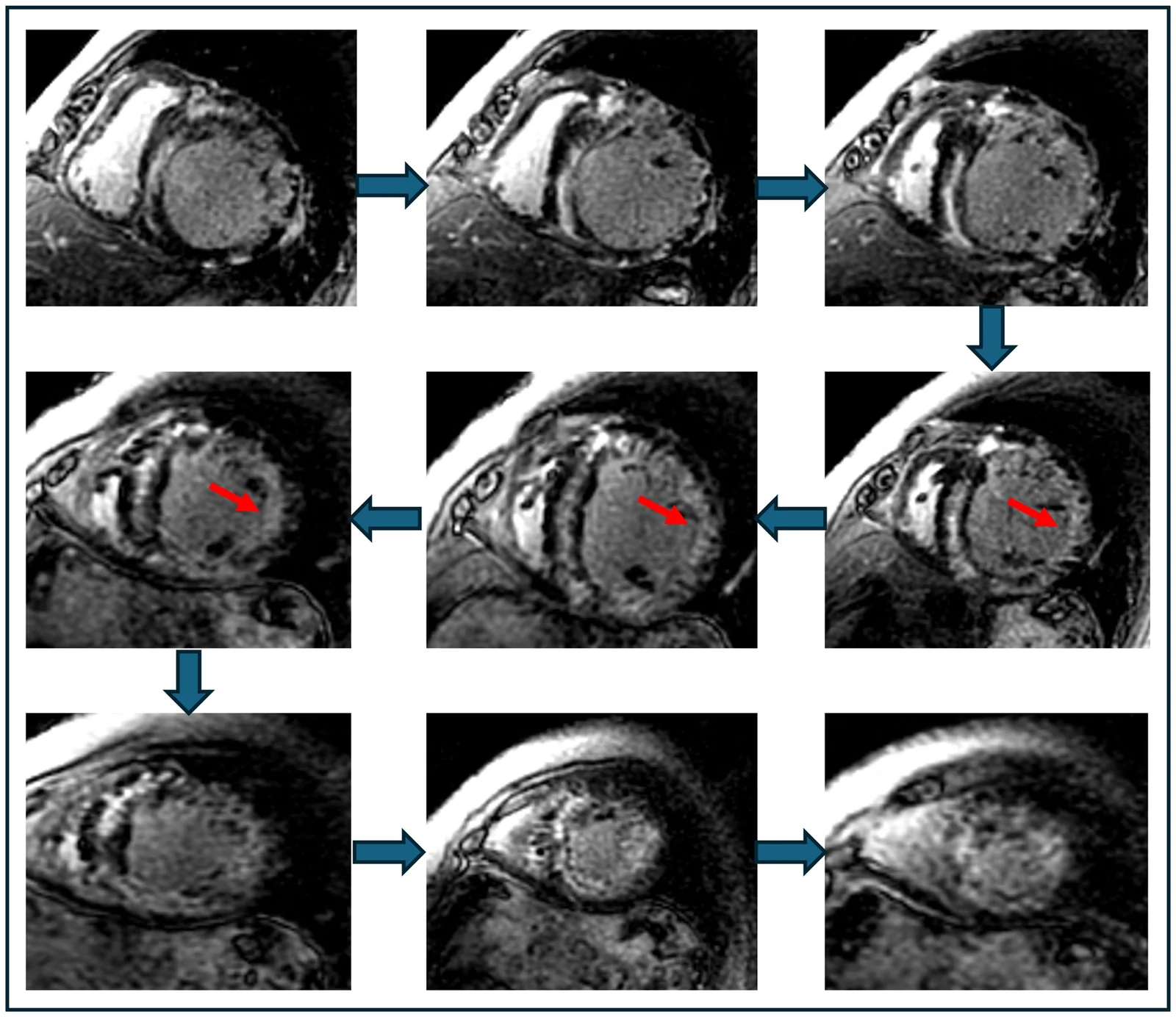
Representative short-axis late gadolinium enhancement cardiac magnetic resonance images from cranial to caudal levels. The images show extensive late gadolinium enhancement in a mixed mid-wall and subendocardial distribution, with areas of near-transmural enhancement. The enhancement was most prominent along the lateral left ventricular wall, as indicated by the red arrows.

The patient was admitted with palpitations for 2 days. On admission, physical examination revealed a continuous LVAD flow murmur, but no peripheral oedema or other signs of decompensated heart failure. Pacemaker interrogation showed several ventricular high-rate episodes occurring over a period of weeks from January to February 2024. Only one documented episode during admission degenerated from VT into ventricular fibrillation (VF) and required external defibrillation. A 12-lead electrocardiogram (ECG) demonstrated wide QRS tachycardia with right bundle branch block morphology and an inferior axis (*Figure 2A*). After brief cardiopulmonary resuscitation, cardioversion was subsequently performed, restoring sinus rhythm with a pacing rhythm.

**Figure 2:**
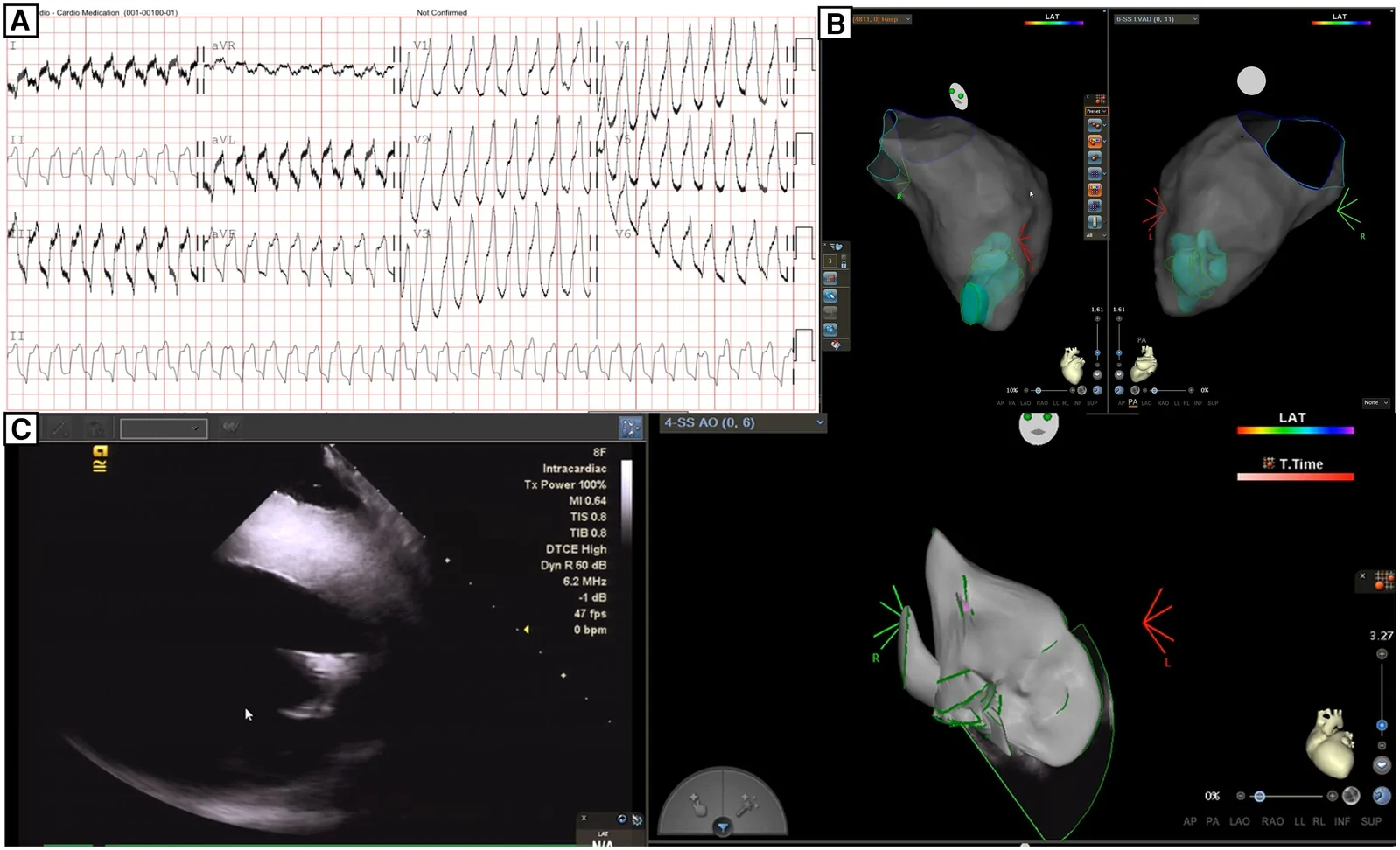
(*A*) Clinically documented ventricular tachycardia with right bundle branch block morphology and inferior axis; (*B*) left ventricle and left ventricular assist device geometry reconstructed using intracardiac echocardiography; (*C*) intracardiac echocardiographic visualization of the left ventricle and left ventricular assist device position.

Following a comprehensive discussion, the patient consented to an electrophysiological study (EPS) and catheter ablation. Intracardiac echocardiography guided the transseptal puncture for the antegrade LV approach, facilitating the creation of LV geometry and delineation of the LVAD positioning (*Figure 2B* and *C*). Left ventricular endocardial mapping was initially performed using an Octaray (CARTO3, Biosense Webster) mapping catheter. Because catheter manoeuvrability around the LV apex was limited by the LVAD, mapping was continued with a DecaNav (CARTO3, Biosense Webster) catheter. Diffuse late potentials were identified from the basal to mid-inferior and lateral walls of the LV (*Figure 3A–C*). Right ventricular extrastimulus pacing induced ventricular premature contraction (VPC) couplets with morphology consistent with the clinically documented VT. During the couplets, the catheter in the mid-lateral LV wall (DecaNav catheter) demonstrated suspected diastolic potential between the QRSs (*Figure 3D*).

**Figure 3:**
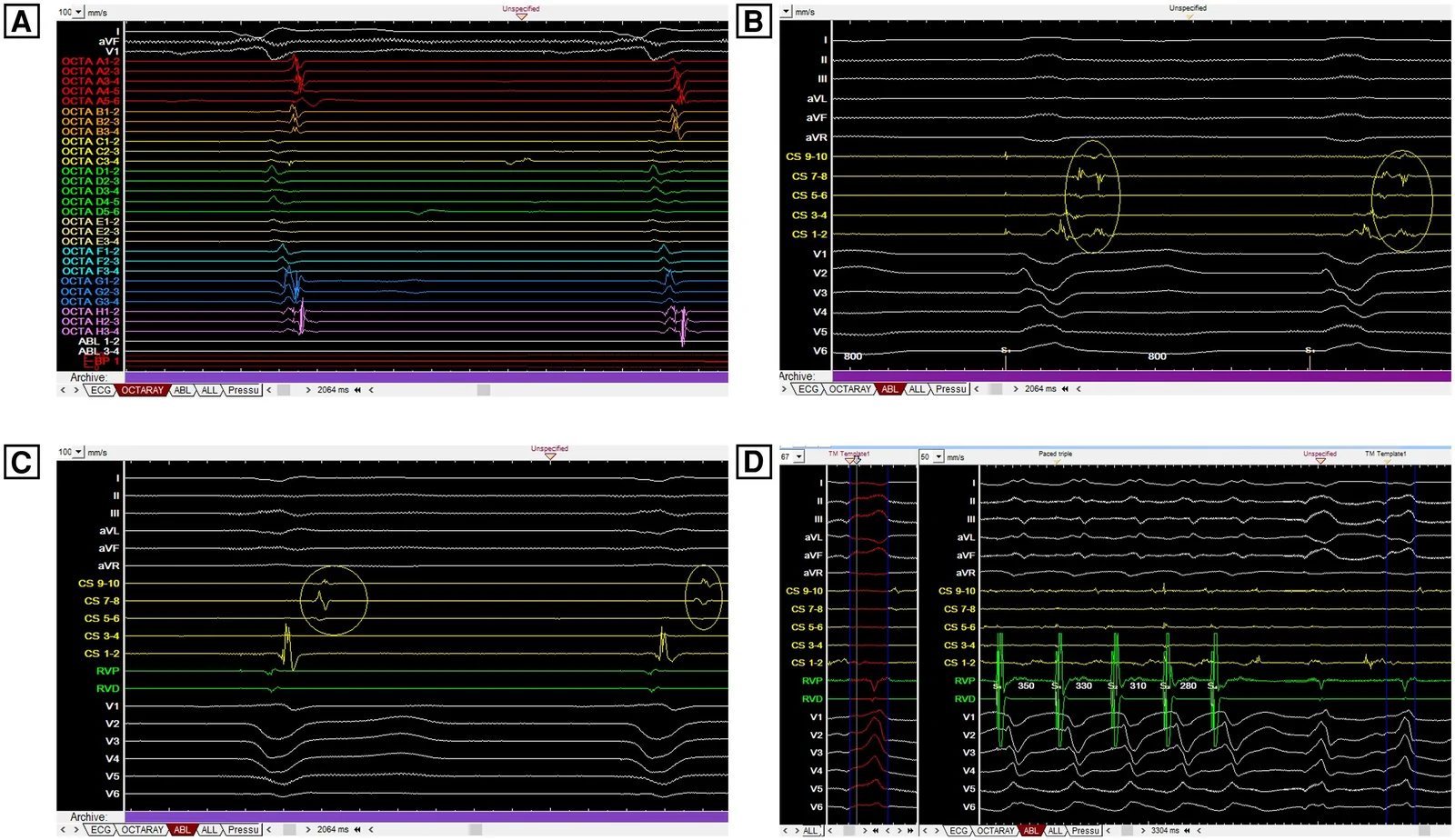
(*A*) Diffuse late potentials recorded over the left ventricular basal–mid-inferior wall using Octaray; (*B*) late potentials recorded along the left ventricular mid-lateral wall using DecaNav; (*C*) late potentials recorded along the left ventricular basal lateral wall; (*D*) right ventricular extrastimulus pacing-induced ventricular premature contraction couplet similar to clinical ventricular tachycardia morphology.

The LV voltage map revealed a low voltage area across the basal to mid-inferolateral LV. Isochronal late activation mapping similarly indicated a deceleration zone within this region (*Figure 4*). Pace mapping was performed at the lateral basal border zone, showing a high PASO correlation of 97% with the clinically documented VT morphology. Consequently, substrate modification was performed around the basal to mid inferolateral LV. Following this intervention, no VPCs were induced by RV extrastimulus pacing. Several months later, CRT-D implantation was arranged, but was unsuccessful due to occlusion of both right and left subclavian veins. The pacemaker interrogation did not detect any VT episode after the procedure. After ablation, the patient continued amiodarone at 200 mg/day along with sacubitril/valsartan and dapagliflozin and remained free of recurrent palpitations and syncope during the 2-year follow-up. Nevertheless, the LVEF declined further to 17% on repeat transthoracic echocardiography, reflecting ongoing progression of the underlying FD despite successful arrhythmia control.

**Figure 4:**
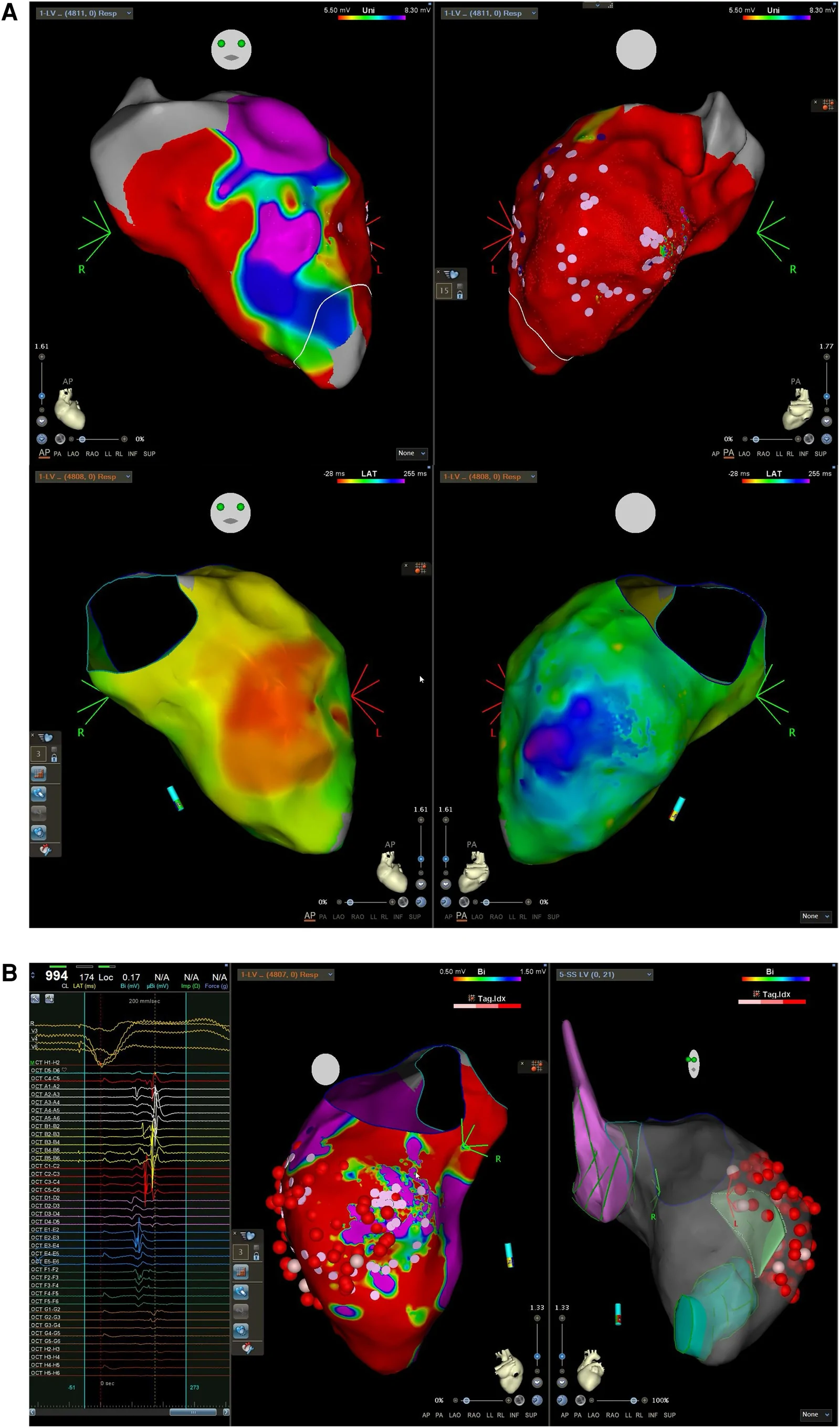
(*A*) Pre-ablation left ventricle unipolar voltage mapping and isochronal late activation maps showed deceleration zones over the left ventricular inferolateral basal–mid-wall; (*B*) substrate modification ablation site around the left ventricular basal–mid-inferolateral wall.

## Discussion

Cardiac manifestations in FD, including hypertrophic cardiomyopathy and arrhythmias, are significant contributors to morbidity and mortality in these patients, requiring targeted therapeutic strategies for both cardiac symptoms and underlying disease pathology. Fabry disease cardiomyopathies could manifest as progressive left ventricular hypertrophy (LVH), extensive myocardial fibrosis, conduction abnormalities, and arrhythmias. Conduction abnormalities, including symptomatic bradycardia and AV block requiring pacemaker implantation, are common in FD. Ventricular arrhythmias are also increasingly recognized and are associated with a significantly increased risk of mortality.^2,6^ It is recommended that FD patients with cardiac involvement must be regularly monitored with 24-h ambulatory ECG monitoring. In patients with late-onset phenotypes, particularly in the presence of extensive myocardial fibrosis, ICD implantation should be considered to prevent SCD due to malignant VA.^3^

Enzyme replacement therapy with agalsidase alfa or agalsidase beta has been a cornerstone in managing FD, aiming to reduce Gb3 accumulation in various organs, including the heart. However, despite early ERT, patients often progress to advanced cardiac disease stages marked by LVH, fibrosis, and arrhythmias, particularly VA. The efficacy of ERT may be limited once significant cardiac fibrosis has developed, as fibrosis is a substrate for VT and poses challenges for management.^3,7^

Ventricular tachycardia ablation in FD has emerged as a feasible, though complex, treatment option, especially in cases resistant to medical therapy and presenting as recurrent VT. Although it has not demonstrated a mortality benefit, particularly in late-stage patients, ICD implantation remains important for preventing SCD. Mills *et al.* reported that the VT mechanism in FD was re-entry around areas of fibrosis. Most of the substrates were transmural, and critical isthmus was found endocardial but also can be found epicardially. With these findings, a combined endocardial/epicardial approach may be helpful for VA ablation in FD.^5,8^ Cardiac magnetic resonance imaging using gadolinium contrast agents plays an important role in visualizing myocardial fibrosis, and previous reports show that this fibrosis is typically distributed in the mid-myocardium of the LV inferolateral wall. The presence of LGE in FD patients correlates with adverse outcomes, including arrhythmias and heart failure, that could serve as a prognostic marker.^9-11^

We reported a case of a patient with late-onset phenotype of FD with progressive cardiac complications, including HFrEF necessitating LVAD implantation and complete AV block requiring a pacemaker. His current presentation included episodes of VT with VF, highlighting the complexity and progression of cardiac involvement in FD. Our EPS findings are comparable to previous reports regarding myocardial fibrosis and arrhythmia substrate distribution in FD patients. Acute arrhythmia suppression was achieved with substrate modification targeting the late potential area. Previous case reports demonstrated that mixed outcomes with long-term survival are often compromised by underlying heart failure and progressive cardiomyopathies. Ventricular tachycardia ablation was performed to enhance a patient’s quality of life; however, its long-term survival benefits are still unsatisfactory.^5^ In advanced FD, LVAD support may provide haemodynamic stabilization in carefully selected patients.^12^

## Conclusion

Catheter ablation may provide acute control of VT, reduce arrhythmia burden, and improve symptom control in patients with FD; however, in advanced FD, its role is best regarded as adjunctive and largely palliative rather than disease modifying. Future directions should focus on early identification and monitoring of cardiac involvement, optimizing ERT timing, and investigating potential adjunct therapies, including substrate reduction or gene therapies, to mitigate progressive cardiac fibrosis and arrhythmogenic potential in FD.

## Lead author biography


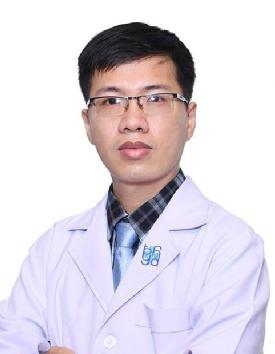
Dr Khuong Dang Tran is a cardiac electrophysiologist with expertise in clinical electrophysiology and cardiac implantable electronic device implantation, including pacemaker therapy. He has ∼5 years of experience in the field of cardiac electrophysiology and is currently working at the University Medical Center Ho Chi Minh City, one of the leading tertiary referral hospitals in southern Vietnam. Dr Tran received his electrophysiology training in Vietnam and further advanced his clinical training at Taipei Veterans General Hospital in Taipei, Taiwan.

## Data Availability

The data underlying this article are available within the article. Additional patient-level data are not publicly available to protect patient privacy but may be made available from the corresponding author upon reasonable request and subject to appropriate ethical approval.
